# Utilizing distributional analytics and electronic records to assess timeliness of inpatient blood glucose monitoring in non-critical care wards

**DOI:** 10.1186/s12874-016-0142-2

**Published:** 2016-04-08

**Authors:** Ying Chen, Shih Ling Kao, E-Shyong Tai, Hwee Lin Wee, Eric Yin Hao Khoo, Yilin Ning, Mark Kevin Salloway, Xiaodong Deng, Chuen Seng Tan

**Affiliations:** Saw Swee Hock School of Public Health, National University of Singapore, National University Health System, Singapore, S117549 Singapore; Division of Endocrinology, Department of Medicine, Yong Loo Lin School of Medicine, National University of Singapore, National University Health System, Singapore, S119228 Singapore; Department of Pharmacy, National University of Singapore, Singapore, S119543 Singapore

**Keywords:** Distributional analytics, Timeliness, Quality of care, Diabetes mellitus, Inpatient, Electronic medical records

## Abstract

**Background:**

Regular and timely monitoring of blood glucose (BG) levels in hospitalized patients with diabetes mellitus is crucial to optimizing inpatient glycaemic control. However, methods to quantify timeliness as a measurement of quality of care are lacking. We propose an analytical approach that utilizes BG measurements from electronic records to assess adherence to an inpatient BG monitoring protocol in hospital wards.

**Methods:**

We applied our proposed analytical approach to electronic records obtained from 24 non-critical care wards in November and December 2013 from a tertiary care hospital in Singapore. We applied distributional analytics to evaluate daily adherence to BG monitoring timings. A one-sample Kolmogorov-Smirnov (1S-KS) test was performed to test daily BG timings against non-adherence represented by the uniform distribution. This test was performed among wards with high power, determined through simulation. The 1S-KS test was coupled with visualization via the cumulative distribution function (cdf) plot and a two-sample Kolmogorov-Smirnov (2S-KS) test, enabling comparison of the BG timing distributions between two consecutive days. We also applied mixture modelling to identify the key features in daily BG timings.

**Results:**

We found that 11 out of the 24 wards had high power. Among these wards, 1S-KS test with cdf plots indicated adherence to BG monitoring protocols. Integrating both 1S-KS and 2S-KS information within a moving window consisting of two consecutive days did not suggest frequent potential change from or towards non-adherence to protocol. From mixture modelling among wards with high power, we consistently identified four components with high concentration of BG measurements taken before mealtimes and around bedtime. This agnostic analysis provided additional evidence that the wards were adherent to BG monitoring protocols.

**Conclusions:**

We demonstrated the utility of our proposed analytical approach as a monitoring tool. It provided information to healthcare providers regarding the timeliness of daily BG measurements. From the real data application, there were empirical evidences suggesting adherence of BG timings to protocol among wards with adequate power for assessing timeliness. Our approach is extendable to other areas of healthcare where timeliness of patient care processes is important.

## Background

Regular monitoring of blood glucose (BG) in hospitalized patients is an important component of inpatient diabetes mellitus (DM) care. The American Diabetes Association (ADA) recommends monitoring blood glucose (BG) four times per day (i.e., before meals and at bedtime) in hospitalized patients with DM. If the patient is fasting or receiving continuous enteral or parenteral nutrition, the recommended BG monitoring frequency is once every 4 to 6 h. BG monitoring is performed every 30 min to 2 hourly, if a patient is on an intravenous insulin infusion [1–4].

Timely measurement of BG facilitates the delivery of inpatient DM care, and allows treatment regimens to be revised to achieve optimal glycaemic control. For example, pre-prandial BG measurement supports clinical decision-making by enabling healthcare providers to prescribe an appropriate dose of supplemental insulin to correct for pre-meal hyperglycaemia. Therefore, a holistic assessment of the quality of inpatient DM care should include an evaluation of compliance to BG monitoring timings. However, methodologies assessing timeliness are not well-established.

A study by Buchs and colleagues assessed the compliance to BG monitoring protocols by using a pie chart to display the proportion of BG timings in a specific portion of time, such as, pre-meals and post-meals [5]. The majority of BG measurements (approximately 75 %) occurred before mealtimes over a 6-month period. However, this method aggregated data over a long period which did not facilitate further evaluation of circumstances surrounding non-adherence, if any was detected. This would limit its utility in a real-world setting.

Continuous evaluation of BG measurements can now be conducted with the advent of electronic medical records [6]. We propose using distributional analytics as a surveillance tool to provide high-resolution empirical evidence of adherence to clinical protocols where timeliness is an important factor [7]. Our proposed analytical approach will address the following: (i) assessment of daily adherence to a BG monitoring protocol by ward over a pre-specified period, and (ii) detection of wards and days exhibiting potential non-adherence or changes in patient care processes.

## Methods

We examined all point-of care (POC) BG measurements performed in 24 non-critical care wards in a 1000-bed tertiary care hospital, National University Hospital, from November to December 2013. Capillary BG measurements were performed using POC glucose meter Accu-Chek Inform II (Roche, Basel Switzerland) and stored in a central laboratory database. The BG data downloaded from the central database contains de-identified patient identifier, BG value, patient location (i.e., ward), date and time of BG measurement. The specialty of the ward indicates the predominant type of patients in the ward. We did not collect additional data including mealtimes and patient demographics, which reflected the current set-up of the laboratory database.

We analyzed all the POC BG timings during the 2-month period. BG timings were converted to hours according to the standard 24-h clock. In the hospital where we conducted the study, the mealtimes are targeted around 0800, 1200 and 1800 h, while the bedtime is targeted around 2200 h.

The majority of inpatients requiring BG monitoring are eating regular meals (i.e., not fasted). Hence, the ideal BG timings should be distributed with multi-modes within a day, where the modes should occur before mealtimes and at bedtime. An extreme contrast would be a uniform distribution in the BG timing, which reflects a complete lack of adherence to ADA-recommended timings for patients eating regular meals. Hence, a simple way to assess potential adherence to BG monitoring protocol is to perform a one-sample Kolmogorov-Smirnov (1S-KS) test that detects deviation of BG timings from a continuous uniform distribution between 0 and 24 h [8], where a significant p-value (i.e., *p*-value < 0.05) suggests potential adherence to protocol. For presentation purposes only, we ranked wards in a decreasing order according to the proportions of days with significant deviations from the uniform distribution over the 2-month period and ties were broken with the median p-value. We plotted the boxplots of p-values for each ward stratified by medical specialty to investigate for potential differences between specialties.

To avoid false negatives with 1S-KS test, we identified wards that are adequately powered by performing a Monte Carlo simulation study to estimate the power for each ward [9–11]. For each ward, we had generated 5,000 simulation iterations for each day. The BG timings were simulated via the inversion of the empirical cumulative distribution function (cdf) on that day and the total number of measurements simulated was the same as the observed number on that day [11, 12]. We used linear interpolation to obtain a continuous cdf for simulation purposes and recorded the simulated power for each day (i.e., the proportion of iterations with *p*-values < 0.05) [13]. The simulation procedure for each BG timing in a day was as follows:

1. We generated a random number from a uniform distribution, i.e., *u* ~ *U*(0, 1)
2. We identified the closest observed BG timings interval, (*t*_1,_*t*_2_], on that day, such that, *F*(*t*_1_) < *u* ≤ *F*(*t*_2_), where *F*(·) is the empirical cdf of BG timings, then ${t}_{simulate}=\left({t}_2-{t}_1\right)\frac{u-F\left({t}_1\right)}{F\left({t}_2\right)-F\left({t}_1\right)}+{t}_1.$

We gathered the estimated power from simulation across all days to compute the median and mean power for each ward. Wards with average or median power more than or equal to 90 % were considered adequately powered for assessing adherence. To understand the interplay among effect size, sample size and p-value across all wards in the hospital, we also regressed –log_10_(*p*-value) of 1S-KS test on standardized effect size and sample size, with interaction between the two standardized quantities. The effect size is the 1S-KS test statistic, $D={ \sup}_y\left|F(y)-\frac{1}{24}y\right|$, where *y* denotes the BG timing and *D* quantifies the magnitude of deviation between the empirical cdf of BG timing and the uniform distribution.

To complete the assessment of BG monitoring protocol with 1S-KS, we corroborated the p-value findings visually with the cdf plots to inspect for features that we would expect from a ward that had been compliant with the protocol. Testing against a uniform distribution is based on a simple assumption of an extreme non-adherence behavior. However, other non-adherent behaviours may exhibit distributions of BG timings that differ from a uniform distribution resulting in statistically significant findings with 1S-KS tests too. To rely less on the parametric assumption of the uniform distribution, we also proposed to test the BG timing distribution of the current day against the previous day via a two-sample Kolmogorov-Smirnov (2S-KS) test [14, 15]. A significant p-value from a 2S-KS test suggests a statistically significant difference in BG timing distributions between two consecutive days.

So far, both the 1S-KS and 2S-KS tests were using BG timings from each day and two consecutive days respectively. By applying mixture modelling on all BG timings in the 2-month period among wards with high power, we can de-convolute the overall distribution of BG timings into components. We assumed a mixture of normal distributions to model daily BG timings where the mean and variance parameters were fixed constants across all days, and we modelled the mixture probability of each component by day where day was a categorical variable. In this finite mixture model, we defined the conditional density as follows:

$$ h\left({y}_{ij}\Big|{\boldsymbol{w}}_{\boldsymbol{ij}},\boldsymbol{\alpha}, \boldsymbol{\mu}, {\boldsymbol{\sigma}}^2\right)={\displaystyle \sum_{k=1}^K{\pi}_k}\left({\boldsymbol{w}}_{\boldsymbol{ij}},\boldsymbol{\alpha} \right)f\left({y}_{ij}\Big|{\mu}_k,{\sigma}_k^2\right), $$

where *y*_*ij*_ denotes the j-th BG timing on the i-th day for *i* = 1, …, 61, and *j* = 1, …, *n*_*i*_ (where *n*_*i*_ is the sample size on the i-th day). Assuming there are K components, *π*_*k*_ is the component probability assigned to the k-th component, and *f*(⋅) denotes the normal density function with component-constant mean, *u*_*k*_, and variance, *σ*_*k*_^2^, where ***μ*** = (*μ*_1_, *μ*_2_, …, *μ*_*K*_) ' and ***σ***^2^ = (*σ*_1_^2^, *σ*_2_^2^, …, *σ*_*K*_^2^) '. We assume a multinomial logit model for the component probabilities with independent variable, ***w***_***ij***_ = (*I*_*ij*2_, …, *I*_*ijd*_, …, *I*_*ij*61_) ', where *I*_*ijd*_ takes a value of 1 if the i-th day is the d-th day in this 2-month period:

$$ {\pi}_k\left({\boldsymbol{w}}_{\boldsymbol{ij}},\boldsymbol{\alpha} \right)=\frac{e^{{\boldsymbol{w}}_{\boldsymbol{ij}}^{\boldsymbol{\hbox{'}}}{\boldsymbol{\alpha}}_{\boldsymbol{k}}}}{{\displaystyle {\sum}_{u=1}^K}{e}^{{\boldsymbol{w}}_{\boldsymbol{ij}}^{\boldsymbol{\hbox{'}}}{\boldsymbol{\alpha}}_{\boldsymbol{u}}}}, $$

where ***α*** = (***α***_***k***_^'^)_*k* = 1,…,*K*_^'^ and ***α***_1_≡0. So the full log-likelihood function for all BG timing in a ward is:

$$ \log L={\displaystyle \sum_{i=1}^{61}{\displaystyle \sum_{j=1}^{n_i} \log }}\left\{h\left({y}_{ij}\Big|{\boldsymbol{w}}_{\boldsymbol{ij}},\boldsymbol{\alpha} \right)\right\}. $$

We used the integrated classification likelihood (ICL) as the criterion for model selection. It is a more robust criterion than Bayesian information criterion (BIC) in the presence of violation in model assumptions [16]. Hence, if the wards with high power were adherent to the BG monitoring protocol, there should be only four components with small standard deviation (SD), which we had arbitrary taken it to be SD < 1, and the mean values of these components should be prior mealtimes and around bedtime. We summarized the mean and variance estimates for each component with SD <1 using the minimum, median and maximum statistics, and plotted the mean ± 1.96 × SD of each component for all wards. For the probability estimates, we first took the average probability estimates within each ward and reported their minimum, median and maximum for each component with SD < 1. For components with SD ≥ 1, their minimum, median and maximum statistics were reported collectively for mean, variance and probability estimates.

We used the R statistical software program to analyze the BG data. R packages, *stats, Matching* and *flexmix* were used to perform the simulation (R functions: *runif* and *approx*), KS-tests (R functions: *ks.test* and *ks.boot*) and mixture modelling (R functions: *stepFlexmix* and *FLXPmultinom*) [14, 17, 18].

## Results

There was a total of 73,182 BG measurements in 23,221 patient-days from 3,673 patients during the period November to December 2013. Among the 23,221 patient-days, 9.4 % of patient-days had more than four BG measurements, 42.3 % of them had four BG measurements, and 48.3 % had less than four BG measurements.

**Fig. 1:**
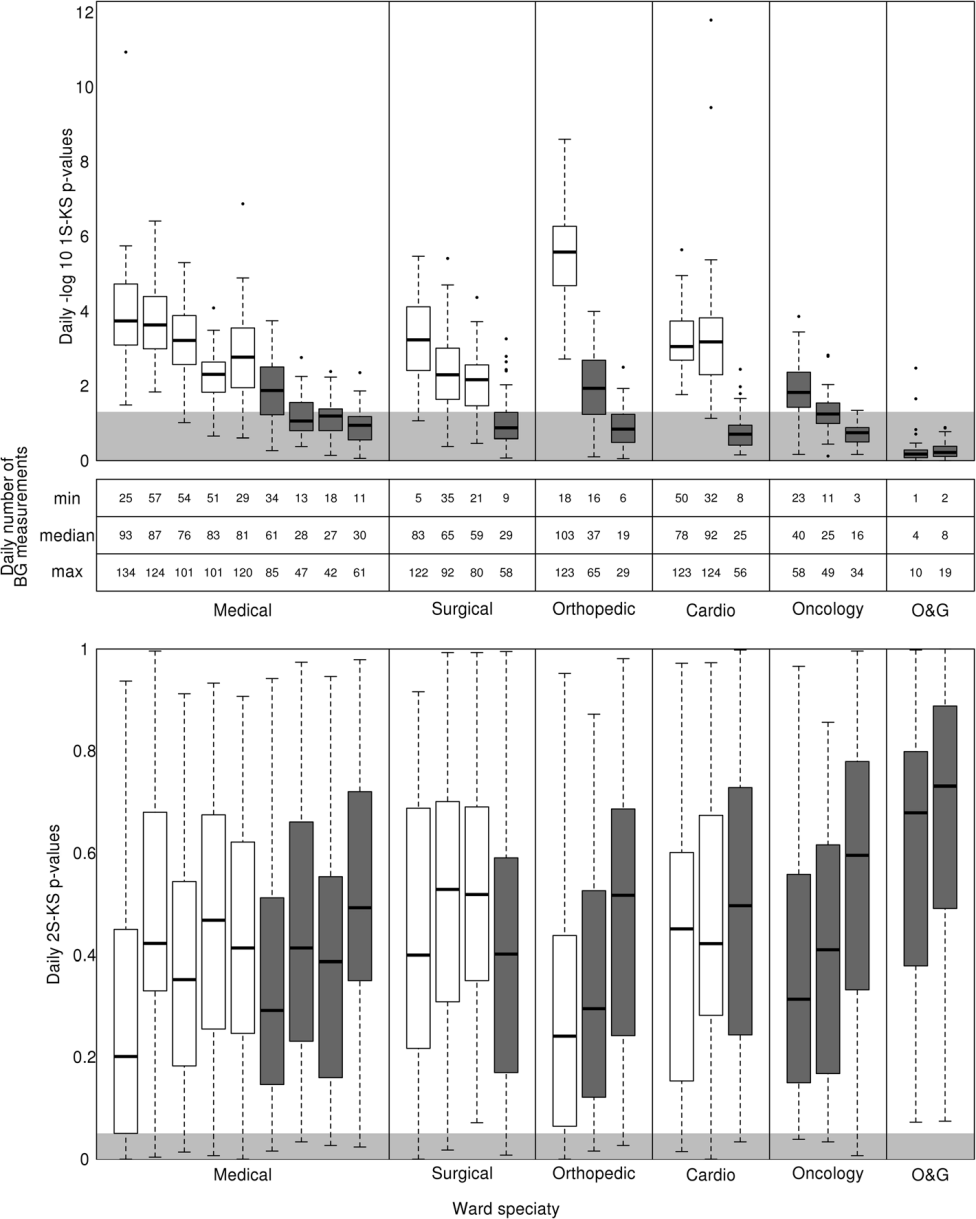
*P*-values of Kolmogorov-Smirnov tests. The *top* panel displayed the boxplots of –log_10_(*p*-values) from the one-sample Kolmogorov-Smirnov (1S-KS), the middle panel displayed the minimum (min), median and maximum (max) daily number of blood glucose (BG) measurements over the 2-month period, and bottom panel displayed the boxplot of *p*-value from the two-sample Kolmogorov-Smirnov (2S-KS) tests. The boxplots were grouped together according to the medical specialties of the wards, where Orthopedic represented Orthopedic surgery, Cardio represented Cardiology, O&G represented Obstetrics and Gynaecology. Boxplots in *white* and *dark gray* shade corresponded to wards with mean or median power ≥ 90 % and <90 % respectively. Within each specialty, wards were ranked by the proportions of significant *p*-values and when there were ties the median p-values were used. The *light gray* region in the *top* and *bottom* panels corresponded to the region where the *p*-values were >0.05 and <0.05 respectively

Figure 1 ordered the boxplots of the 1S-KS test p-values for all 24 non-critical care wards by the proportions of days with significant p-values within each specialty. The boxplots with dark gray shading corresponded to wards that were not adequately powered and these wards were either small wards, or obstetrics & gynecology (O&G) wards that had much fewer patients on BG monitoring. Wards with high power had median sample size ranging from 59 to 103 BG measurements in a day. When we examined the relationship of the p-values with sample size and effect size across the 24 wards, a one unit increase in the standardized sample size alone could lead to an increase of 2.56 in –log_10_(*p*-values) keeping standardized effect size fixed at 1, which is about 99.7 % reduction in p-values, and similarly a one unit increase in the standardized effect size alone could lead to an increase of 2.41 in –log_10_(*p*-values) keeping standardized sample size fixed at 1, which is about 99.6 % reduction in p-values (see Table 1). Hence, the change in p-value that was attributable to sample size and effect size respectively was comparable.

**Table 1:** The relationship of –log_10_(*p*-values) with effect size and sample size

	Beta (95 % CI)
Standardized effect size	1.52 (1.5, 1.54)
Standardized sample size	1.67 (1.66, 1.69)
Interaction between the two standardized quantities	0.89 (0.87, 0.9)
Coefficient of determination (*R* ^2^)	97.2 %

**Fig. 2:**
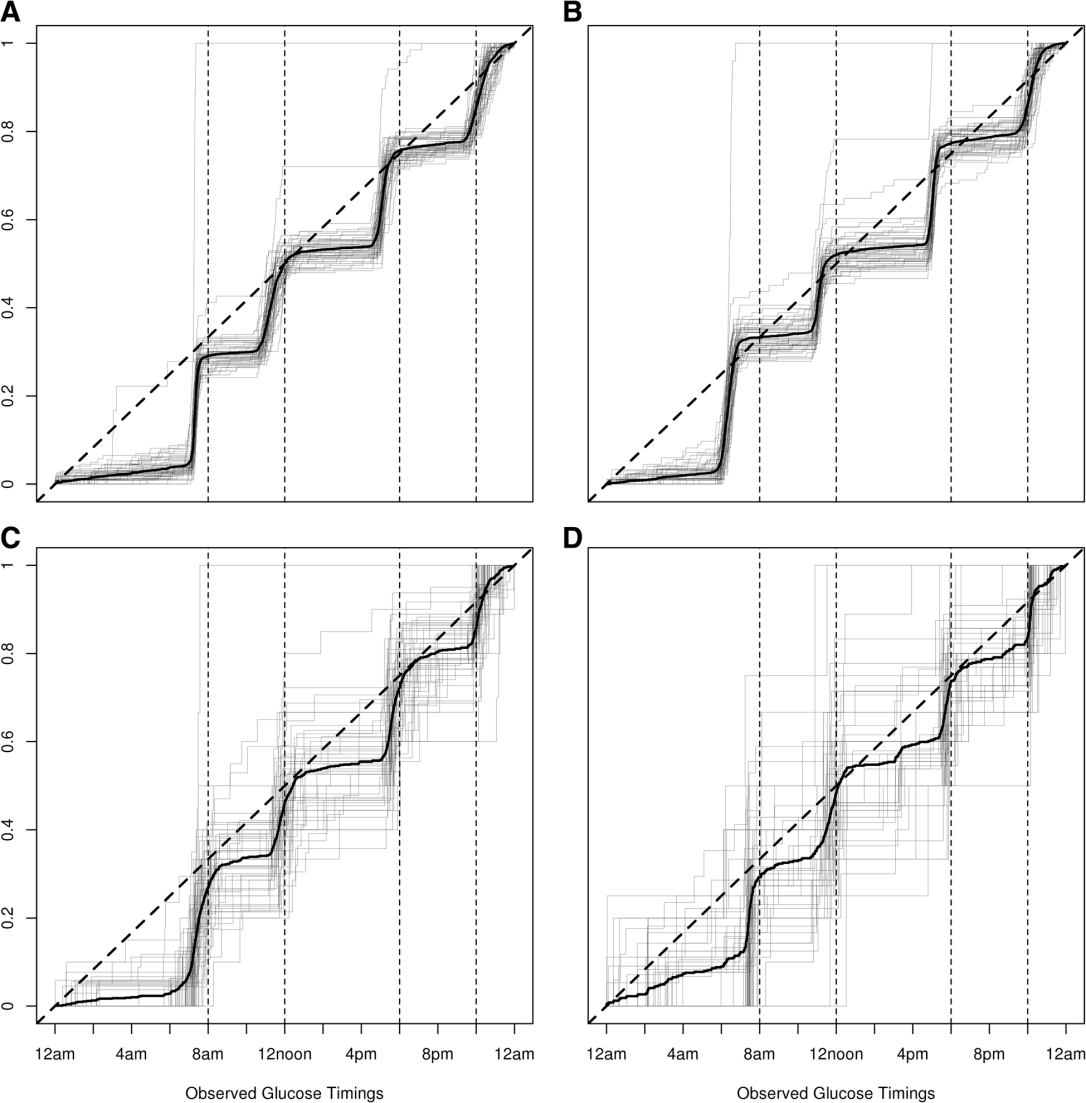
Cumulative distribution function plots of daily BG timings for selected wards. Panels **a** and **b** displayed the top two highly ranked wards using the one-sample Kolmogorov-Smirnov (1S-KS) test in Fig. 1; panels **c** and **d** display the two lowest ranked wards. Each of the solid line was a cumulative distribution function (cdf), where the *light grey* lines represented cdfs of daily BG timings and the *dark gray* lines represented cdfs of aggregated BG timings over the 2-month period. The *diagonal dotted* lines represented the cdf of a uniform [0, 24], i.e., the reference distribution used in the 1S-KS test

A significant 1S-KS test p-value only suggests the particular day is potentially adherent to protocol. To ascertain the adherence status of wards, we corroborated the p-values with daily cdfs of BG timings. For the two highly ranked wards (i.e., Rank 1 and 2 in Fig. 2a and b respectively), we found that the majority of days had exhibited four pronounced steps before mealtimes and around bedtime suggesting adherence to BG monitoring protocols. This was also observed in the other wards with high power. There were only a few days that had fewer pronounced steps than the ideal BG timing distribution. When we further explored the days with fewer pronounced steps, we found that there were no BG records on prior days suggesting potential missing data influencing the BG timing distribution. The prevalence of days without any BG measurements were low. There were 31 instances where no BG measurements were available on an entire day among the 11 wards with adequate power in the 2-month period (i.e., a total of 671 instances = 61 days × 11 wards) and 27 instances among 13 wards with inadequate power in the same period (i.e., a total of 793 instances = 61 days × 13 wards). For the two lowest ranked wards with low power (i.e., Rank 23 and 24 in Fig. 2c and d respectively), the lines were more jagged and less pronounced when compared to wards with high ranks. The wards with low ranks were O&G wards and patients from these wards were mostly fasting and hence monitored more frequently in every 4–6 h.

In Fig. 1, the majority of 2S-KS test p-values were insignificant. For the scenarios where two consecutive days had significantly different BG timing distributions, they contained the days with less than four pronounced steps observed in Fig. 2 previously. Further exploration among wards with high power by utilizing both the 1S-KS and 2S-KS *p*-values within a two consecutive days moving window did not suggest frequent potential change from or towards non-adherence to protocol (i.e., a significant 2S-KS p-value, and only one significant 1S-KS *p*-value) as only three occurrences across all wards over the 2-month period had transitions from non-adherence to adherence or vice versa.

**Fig. 3:**
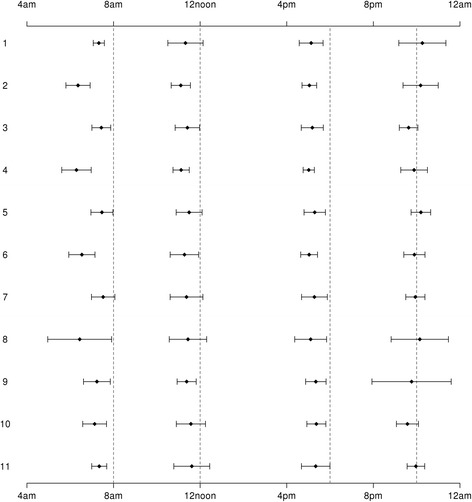
The four components from mixture models with small standard deviations among wards with high power. The four components were ordered by their mean estimates. *Diamonds* represented the mean estimates and the solid horizontal lines with ticks at the two ends represented the ±1.96SD widths. For the component corresponding to before breakfast, only ward with Rank 7 crossed 8 am; for the component corresponding to before lunch, wards with Rank 1, 5, 7, 8, 10 and 11 crossed 12noon; for the component corresponding to before dinner, no wards crossed 6 pm; for the component corresponding to bedtime, all wards crossed 10 pm

The daily BG timings were expected to be distributed with four modes, i.e., before mealtimes and at bedtime, if the BG monitoring protocol was being adhered to. Among the wards with high power (≥90 %), there was evidence suggesting adherence to protocol. Hence, we modelled each ward individually using mixture models which de-convoluted the BG timing distribution into components. From the mixture modelling analysis, we consistently identified four components with SD estimates < 1. In Fig. 3, we visualized the four components identified for each ward with their mean and the interval corresponding to mean ± 1.96SD (indicating 95 % of the BG measurements were within this interval). Most of the wards were adherent to protocol because the majority of their measurements (i.e. ≥ 95 %) were taken before mealtimes (except lunch time) and around bedtime.

Among wards with high power, we found that the mean estimates of the component corresponding to before breakfast time, where the expected breakfast time was 8 am, the median of the mean estimates was about 46 min earlier than 8 am, and it ranged from 103 to 29 min earlier than 8 am (Table 2). For the component corresponding to before lunch time, where expected lunch time was 12noon, the median of the mean estimates was about 37 min earlier than 12noon and it ranged from 53 to 23 min earlier than 12noon. For the component corresponding to before dinner time, where the expected dinner time was 6 pm, the median of the mean estimates was 49 min earlier than 6 pm and it ranged from 59 to 38 min earlier than 6 pm. For the component corresponding to bedtime, where the expected bedtime is 10 pm, the median of the mean estimates was just 4 min before 10 pm and it ranged from 26 min before 10 pm to 16 min after 10 pm. The range of the mean estimates was the largest for the component corresponding to before breakfast time and it was almost twice as large when compared with the remaining three components.

**Table 2:** Mean, standard deviation and mixture probability estimates from the mixture models among wards with high power

	Components with SD < 1				Components with SD ≥1
	First	Second	Third	Fourth	Others
Mean (converted to hours according to 24-h clock)	7.23 (6.29, 7.52)	11.38 (11.11, 11.61)	17.18 (17.02, 17.37)	21.94 (21.57, 22.26)	11.22 (4.38, 21.56)
Standard deviation (hours)	0.28 (0.13, 0.75)	0.34 (0.19, 0.44)	0.25 (0.13, 0.38)	0.26 (0.21, 0.93)	5.94 (1.58, 6.55)
Mixture probabilities	0.22 (0.18, 0.29)	0.18 (0.16, 0.2)	0.22 (0.19, 0.23)	0.2 (0.15, 0.23)	0.12 (0.06, 0.22)

Median (minimum, maximum) were reported for mean and standard deviation (SD). For mixture probabilities, we first took the average estimates within each ward and reported the median (minimum, maximum)

As for the SD estimates, the median across the four components were between 0.25 and 0.34 h (i.e., for each component, 95 % of the BG measurements were approximately within an hour, or an hour and 20 min interval). For components with SD ≥ 1, the range of the total number of components were 1 and 3. These components may potentially represent the BG measurements taken from patients with hypoglycaemia, or patients who were fasting or receiving insulin infusions. The minimum SD for these components was around 1.6, suggesting 95 % of the BG measurements were within a time interval ≥ 6 h. The majority of the probability estimates for each component were distributed almost equally across the components, except for a few cases where the components with large SD had smaller probabilities when compared to the four components with small SD.

## Discussion

In this paper, we applied distributional analytics-based methodology, mixture modelling and visualization to assess adherence to an inpatient BG monitoring protocol in non-critical care wards. To alleviate the occurrence of false positives due to the interplay of small sample size and effect size in the 1S-KS test, we performed a simulation study to identify wards where the daily total number of BG measurements had a mean or median power greater than or equal to 90 % over the 2-month period (i.e., wards with high power). Restricting our assessment to these wards, we further corroborated the 1S-KS test findings using: (1) visualizations with cdf plots, (2) 2S-KS tests, and (3) mixture modelling.

The BG timing distribution of wards with high power exhibited four modes before three mealtimes and around bedtime. From the cdf plots, we observed four pronounced steps and from the mixture modelling, we observed exactly four components with small standard deviation. This phenomenon concurred with our expectation of a ward following ADA monitoring recommendations for patients eating regular meals. In particular, the cdf plots and the mean ± 1.96SD intervals from mixture modelling allowed us to identify the time interval where majority of BG measurements was taken, which was not possible using the pie chart with aggregated BG data [5]. These visualizations facilitate further investigations of non-adherence by healthcare administrators by identifying problematic timings during the day. This highlights the importance of pairing statistics with visualization to deliver actionable information. When we combined both the 1S-KS and 2S-KS tests within a 2-day moving window over the 2-month period, there was a low occurrence of change from or towards non-adherence to protocol.

Although we could only draw conclusions for wards with high power to minimize false negatives, we could accrue a sufficient number of BG timings for those wards with moderate power by aggregating the BG data over a two-day window. For example, the 6-th medical ward in Fig. 1 had median power close to 90 % (i.e., 87.3 %) and median effect size close to the wards with high power. We proposed to combine the BG timing of the current day of interest with its previous day to obtain a larger sample size while assuming the patient care process was the same between two consecutive days. With a two-day window, we obtained six other wards with an average or median power greater than 90 %, and only the last ward in the first five specialties and the two O&G wards in Fig. 1 were not adequately powered. The results on adherence for these six additional wards with moderate power were similar to the 11 wards with high power. In particular, we identified 20 out of 360 instances = 60 2-day windows × 6 wards were potentially non-adherent to BG protocol and there were no occurrence of change from or towards non-adherence to protocol.

As our proposed approach uses ward level as the unit of analysis, it captures the correlation of BG timings within a patient through the marginal distribution of BG timings from all patients in the ward. Although we had identified some days without BG measurements, these occurrences were low and sporadic, and therefore unlikely to affect our findings.

Our proposed approach provided an analytical way to alert healthcare administrators of potential non-adherence to protocol for a specific day and ward. However, to extend our approach to continuous surveillance through daily monitoring, we proposed to modify the criterion for wards with high power based on the estimated power for each day instead of the mean or median power over the entire 2-month period. This will be useful for a large hospital and will open up the opportunity to monitor adherence on a daily time-scale.

## Conclusion

In this paper, we have proposed a way to assess adherence to BG monitoring protocols using electronic BG records. To reduce false negatives from our proposed assessment with 1S-KS test, we used concepts from power calculation and simulation to determine the wards that are adequately powered with empirical data. By investigating the p-values of KS tests, cdf plots and mixture modelling, we found that wards with high power were adherent to the BG monitoring protocol.

In summary, our approach leverages on the distributional analytics and the availability of electronic records of laboratory data to provide a practical surveillance tool for identifying potential non-adherence to clinical workflow. Our approach is also applicable to other areas of healthcare where timeliness of patient care processes is paramount, for example, medication administration or timed blood investigations.

### Ethics approval and consent to participate

The investigations were carried out in accordance with the ethical codes and guidelines of the Nuremberg Code (1946), the Declaration of Helsinki (1964), the Belmont Report (1979) and the Singapore Guideline for Good Clinical Practice (1998), and were approved by the National Healthcare Group Domain Specific Review Board, including waiver of informed consent.

### Consent for publication

Not applicable.

### Availability of data and materials

The dataset supporting the conclusions of this article is available in: http://blog.nus.edu.sg/dasa/bgdataset1.

## Abbreviations

1S-KS: one-sample Kolmogorov-Smirnov
2S-KS: two-sample Kolmogorov-Smirnov
ADA: American Diabetes Association
BG: blood glucose
BIC: Bayesian information criterion
Cdf: cumulative distribution function
DM: diabetes mellitus
ICL: integrated classification criterion
O&G: obstetrics & gynecology
POC: point-of-care
SD: standard deviation
